# Spheroids and organoids derived from colorectal cancer as tools for *in vitro* drug screening

**DOI:** 10.37349/etat.2024.00226

**Published:** 2024-04-25

**Authors:** Sahira Syamimi Ahmad Zawawi, Elyn Amiela Salleh, Marahaini Musa

**Affiliations:** University of Southampton, UK; Human Genome Centre, School of Medical Sciences, Universiti Sains Malaysia, Kubang Kerian 16150, Malaysia

**Keywords:** Organoid, colon cancer, drug screening

## Abstract

Colorectal cancer (CRC) is a heterogeneous disease. Conventional two-dimensional (2D) culture employing cell lines was developed to study the molecular properties of CRC *in vitro*. Although these cell lines which are isolated from the tumor niche in which cancer develop, the translation to human model such as studying drug response is often hindered by the inability of cell lines to recapture original tumor features and the lack of heterogeneous clinical tumors represented by this 2D model, differed from *in vivo* condition. These limitations which may be overcome by utilizing three-dimensional (3D) culture consisting of spheroids and organoids. Over the past decade, great advancements have been made in optimizing culture method to establish spheroids and organoids of solid tumors including of CRC for multiple purposes including drug screening and establishing personalized medicine. These structures have been proven to be versatile and robust models to study CRC progression and deciphering its heterogeneity. This review will describe on advances in 3D culture technology and the application as well as the challenges of CRC-derived spheroids and organoids as a mode to screen for anticancer drugs.

## Introduction

Currently, only a small percentage (10%) of new anticancer drug candidates to be enrolled in phase I trials are eventually approved by the Food and Drug Administration (FDA) [1]. Compared to other therapeutic areas, cancer has the lowest approval rate of new drugs [2]. A major hurdle in developing new anticancer agent is the transition from the laboratory to the clinic [3]. Experimental models have been a key element in cancer research. However, many cancer models lack the ability to recapitulate the complex and heterogeneous nature of human tumors thus limiting further understanding on carcinogenesis and response to treatment [4].

The establishment of cell culture, developed in the early 19th century [5], has asserted its importance in cell biology research including analysis on cell behavior or response toward external stimuli such as drug through two-dimensional (2D) monolayer cell culture. However, this technique is limited by its inability to mimic the heterogeneity of original cancer [6]. More advanced technology such as three-dimensional (3D) spheroid and organoid culture has been proposed to be the preferred pre-clinical *in vitro* tools to study cancer progression and drug efficacy.

Organoid technology which was initially described in gastrointestinal (GI) organs, allows long-term expansion of normal and cancer tissues without genetic alterations. Despite extensive serial sub-culture *in vitro*, organoids from non-transformed tissues demonstrated genetic and epigenetic stability when compared to cell lines [7–9].

Spheroids and organoids are being applied to unravel the molecular and genetic properties of colorectal cancer (CRC) initiation and development [10–12]. CRC, which arises from accumulation of genetic and epigenetic aberrations is a highly heterogeneous entity. This intratumor and intertumor heterogeneity of CRC may influence treatment efficacy and response to anticancer agents and subsequently will affect patient survival [13, 14]. 3D cultures derived from CRC patients consist of phenotypically heterogeneous and interchangeable spheroid-forming cells with different growth rates and drug sensitivity [15]. Interestingly, organoids derived from CRC and metastatic tissues also showed preserved genetic diversity and morphological stability [16, 17]. Apart from CRC, tumor-derived organoids have been established in several cancer types, including breast, bladder, lung, pancreatic, and prostate cancer [16, 18].

In view of the growing evidence of spheroid and organoid as promising tools in oncology, the paper will address important characteristics for successfully constructed 3D cultures derived from CRC and outlines their latest applications, in addition to their advantages over other *in vitro* methods as well as their limitations to screen for drugs as treatment for CRC.

## Spheroid and organoid: definition and properties

Spheroids and organoids are often used interchangeably due to similarities in their properties although many have described the differences between these 3D culture entities. According to Fennema et al. [19], spheroids refer to simple clusters of a broad range of cell types including tumor spheroids, embryoid bodies, hepatospheres, neurospheres, and mammospheres. Typical sources for spheroids are cancer cell lines or dissociated cell clusters from tumor mass in nonadherent substrates [20]. Due to adherent cells’ tendency to aggregate into higher cell densities, 3D spheroids have been widely used as multicellular models *in vitro*. This model allows for abundant chemical and mechanical interactions, and these were useful in investigating various *ex vivo* tissue models [21]. Generally, spheroids comprise of a single cell type are referred to as homotypic spheroids, while those consisted of multiple cell types are called heterotypic spheroids [22]. Heterotypic spheroids consisting of cancer cells and stromal cells of the tumor microenvironment (TME) namely cancer-associated fibroblasts (CAFs) are widely used for drug discovery [22, 23].

The word "organoid" has been proposed and described by many prominent scientists in the respective field [24, 25]. An organoid refers to a self-organized 3D structure, grown from populations of organ-specific cells derived from sources including pluripotent stem cells (PSCs), adult stem cells (ASCs), or somatic cells of human tissues such as cancer cells. Organoids mimic complex key structure, function, and biology of organs or tissues from which they are derived [26]. The cells exhibit extended proliferation and differentiation capabilities over a long-term of culture, depending on specific culture medium used, and possess certain characteristics reminiscent of the organ or tumor from where they derived from [27–29]. According to Velasco et al. [30], a large portion of organoid model protocols employed stem cells as the cellular source for organoid generation despite that it can be produced from shredded tissue of epithelial cells. Despite having lower cell types compared to PSC-derived organoids, 3D cultures from ASCs can be coaxed to recapitulate the histology and genetics of their parental tissues [31].

Development of spheroid and organoid have been groundbreaking in deciphering human development, disease initiation, and progression as well as the personalized medicine approaches which may not be possible with animal models. The application of 3D culture is an advanced method, particularly in studying cancers and discovering potential anticancer drugs for cancers including CRC.

## CRC

CRC, a complex neoplasm originating from large intestinal epithelium is the third most common cancer which ranks the second in cancer-related mortality worldwide [32]. It is known as a disorder of the elderly predominantly, although there is increasing incidence among those younger populations [33]. Approximately one third of CRC cases account for inherited type which include hereditary non-polyposis colon cancer (HNPCC) syndrome, inflammatory bowel disease (IBD), and familial adenomatous polyposis (FAP). Majority of CRC cases are detected in developing countries with prone for adaptation into unhealthy and Westernized lifestyle [34]. The malignant transformation generally involves a sequential accumulation of various genetic, and epigenetic modifications with consequent deregulation of signalling pathways essential for cancer invasion and metastasis such as wingless/integrated (Wnt)/β-catenin and transforming growth factor beta (TGF-β)/suppressor of mothers against decapentaplegic (SMAD) [35].

CRC is a highly complex and heterogeneous disease, contributed by cancer epithelial cells and the TME [36]. TME constitutes multiple cellular components neighbouring to the colonic crypt of cancer epithelial cells including fibroblasts, immune cells, and endothelial cells and intricate network of extracellular matrix (ECM) which have been reported to promote colorectal carcinogenesis and confer chemoresistance [37].

**Figure 1:**
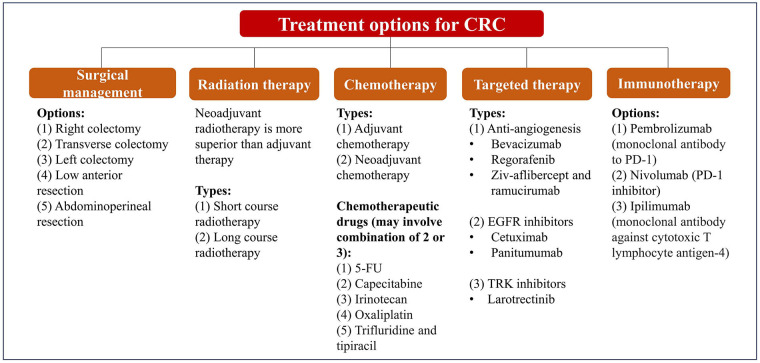
Summary of conventional treatment and management of CRC. 5-FU: 5-fluorouracil; EGFR: epidermal growth factor receptor; TRK: tropomyosin receptor kinase; PD-1: programmed death 1

There are many available options of treatment for CRC which depend on the severity [metastatic *vs.* non-metastatic CRC (mCRC)], molecular type and staging of the disease, as summarized in Figure 1 [38–42]. Traditionally, surgery is applied to control and treat CRC, and it can be performed with other modes of therapies such as chemotherapy, radiation therapy, and targeted therapy.

The complex and heterogeneous nature of CRC has imposed a challenge towards refinement in the profiling and treatment of CRC. Hence, CRC patients remain afflicted with poor prognosis and relapse especially those with overt metastases which are limited to conventional chemotherapy as their only curative option [43]. These lines of chemotherapy include 5-FU are commonly used in combination with folinic acid, irinotecan, or oxaliplatin. The use of fluoropyrimidines in 5-FU, have demonstrated efficacy in the tumor growth control compared to the single-use of fluoropyrimidines alone, owing to the synergistic effects exerted from the combination of 5-FU and oxaliplatin [44]. Accumulating evidence further indicates that 5-FU/folinic acid with oxaliplatin are superior in the treatment of advanced CRC cases compared to 5-FU alone [45]. However, their innate resistance is typically noted in 90% of patients with metastatic cancer and becoming increasingly prevalent with a morbidity rate at 12.5% [46]. In addition, common monoclonal antibodies against CRC, namely cetuximab, bevacizumab, and panitumumab have been increasingly recognized as poorly inefficient and ineffective [47, 48]. The inherent chemoresistance developed by CRC tumors on this line of chemotherapy which is practically still the mainstay of CRC treatment further attributes to the increasing morbidity and mortality.

Given that, considerable efforts have been made over the past decades through cancer modelling *in vitro* for better prediction in drug response. The use of conventional 2D *in vitro* system has greatly provided numerous insights related to tumor progression and therapeutic sensitivity. Yet, number of drugs proven active in 2D *in vitro* and parallel with *in vivo* and clinical studies are too few [49].

## *In vitro* drug screening methods for CRC

Key preclinical stages of drug discovery start with initial target identification and validation, through assay development, high throughput screening, hit identification, lead optimization, and lastly the selection of a candidate molecule for clinical development [50]. *In vitro* pre-clinical drug screening methods with inclusively highlighted 3D spheroids and organoids still pose great advantages despite several drawbacks which have been reported (Table 1). The following section will describe various *in vitro* techniques for early drug discovery and screening for CRC.

**Table 1:** Advantages and disadvantages of *in vitro* methods for preclinical drug screening for CRC

**Methods**	**Advantages**	**Disadvantages**	**Key types**	**References**
2D cell culture system	Simple and low consumption for maintenance; rapid screening; high reproducibility	Require considerable large panel; marked discrepancy with *in vivo* and clinical studies; lacks *in vivo* characteristics	NCI60 panel	[51, 52]
			Bodmer group (> 120 CRC cell lines)	[53]
			Co-culture of CRC cell lines with CAFs	[54, 55]
Microfluidic based-cell culture system	Low reagent consumption; allow high throughput screening; stable biochemical/concentration gradient; shear stress generation; microvasculature	Lack of representative functional vascular network and heterogeneous phenotype of tumor; present transient effects of cell-drug behavior; not able to withstand long-term experiment; difficult to determine individual cell effect	Microfluidic CRC culture	[56–59]
			Microfluidic co-culture of CRC with fibroblasts	[60, 61]
3D spheroid	(1) Recapitulate tumor oxygen gradients and hypoxia with multiple layers of formation (2) Retain chemoresistance behaviour with multiple layers and decreased sphericity (3) Recapitulate the multifaceted and heterogeneous TME with multiple cells culture	(1) Avascular characteristic and passive chemical diffusion disrupt cells processes or functions and affect drug readout (2) Lack of size uniformity leads to data variation (3) Complex and non-standardized drug readout assays (4) Difficult to determine individual cell effect to drug	Scaffold-free spheroid	[23]
			Co-culture CRC spheroids with fibroblasts	[60, 62–64]
			MCTS	[65]
3D organoids	(1) Withstand long-term culture; recapitulate tumor heterogeneity with heterogeneous drug responses; reproduce molecular and cellular composition of tumor (2) Recapitulate tumor ECM stiffness; support the organoids growth and sensitivity to drug (3) Retain genomic characteristic of tumor (4) Retain inter-tumor heterogeneity; support personalized therapy (5) Specific cancer subtype modelling for precision medicine	(1) Time-consuming; laborious; inefficient data analysis; intra- and inter-batch heterogeneity; low batch-to-batch reproducibility (2) Lack of cells intrinsic processes such as cell adhesion and migration can lead to inefficient drug readout (3) Limited predictive ability to certain drug response (i.e., combination based involving oxaliplatin response)	Organoids in Matrigel	[66]
			Organoids in chemically defined synthetic hydrogels	[67]
			CTCDO	[68]
			PDO	[69–72]
			Serrated CRC organoids	[73–77]

NCI: National Cancer Institute; CTCDO: circulating tumor cells (CTCs)-derived organoids; MCTS: multicellular tumor spheroids; PDO: patient-derived organoid

### 2D cell culture system

Cell lines have been used extensively to study the origin of CRC as well as to discover new therapy avenues for the disease. Although animal model has been used to overcome some of the limitations of 2D culture, the failure rate of new anticancer drugs in clinical trials remains significantly high, since animal study performed *in vivo* does not reflect the human physiology [78, 79]. Moreover, the use of animal models has become impractical in most cases where multiple drugs and wide range of concentration of each drug are to be tested [80].

In the context of cancer, drug response is strongly correlated with the genetic and epigenetic makeup of cancer [81]. Hence, the application of cell culture is a relevant laboratory approach where tissue-derived cell lines are grown and maintained in a controlled environment outside of living organism (i.e., *in vitro*), allowing for disease modelling including cancer and identification of active compounds or drugs with anti-cancer properties *in vitro* [82–84].

The extensive application of 2D cell culture for drug screening also includes co-culture which aim to cater the lack of crosstalk between different cells within the monolayer system. For anti-cancer drug screening model, co-culture studies provide closer inspection of TME via interaction with cancer epithelial cells and other cellular components of the TME such as CAFs (activated fibroblasts) and endothelial cells that could influence cancer cells behaviors to chemotherapeutic agents [85–87]. Significant crosstalk has been illustrated between isolated CAFs and CRC cell lines (HCT116 and LoVo) in mediating colorectal carcinogenesis and cisplatin resistance [54]. Loss of 5-FU sensitivity was demonstrated in the co-culture of CAFs and HCT116 and DLD-1 cells [55]. Through co-culture system, CAF role in mediating chemoresistance was demonstrated, providing a drug screening platform.

Despite the efforts to ameliorate lack of cell-cell interaction in the 2D monolayer, the 2D co-cultured cells were similarly affected with limited *in vivo* characteristics. Moreover, the growth rate of the co-cultured cells that may vary between different cell types and at different passages [82]. Additionally, 2D system has poor control of its culture medium substrate composition, seeding density, and other culture conditions that can negatively affect the results [88, 89]. 2D tumor models often exhibit changes in cell morphology and functions different to *in vivo* studies [90]. It is therefore necessary to develop *in vitro* CRC models with higher physiological relevance to better predict anti-cancer drug response.

### Microfluidics-based cell culture system

Considering the limitations of conventional 2D cultures, microfluidics technology has garnered the attention for its microculture system with the advantage of being cost-effective, low consumption of reagent, and providing the platform for high-throughput drug screening [91]. Several *in vitro* drug screening platforms or models can be established through microfluidics technology, namely microfluidics-based models and these include cell-on-a-chip, tissue-on-a-chip, and organ-on-a-chip by incorporating the 2D or 3D cell culture systems [92].

The microfluidics-based culture system offers precise drug screening through the characterization of cell-drug behavior based on several important aspects that correspond to chemotherapeutic mechanism. This includes the stable biochemical gradient generated from molecules circulating such as nutrients and oxygen that are in gradients within the tumors and greatly affect cancer progression and therapeutic efficacy [93]. Besides, microfluidic-based models can mimic the fluid shear stress generated from continuous supply and flow of culture medium with a peristaltic or syringe pump. In the context of CRC, flow shear stress causes dramatic decline of CRC cell lines growth [56]. Improved resistance to clinically relevant doses of combined 5-FU and oxaliplatin was demonstrated in both HCT116 and HT29 tumoroids grown in perfused microenvironment of gravitational microfluidic [57]. Another key feature of microfluidic-based models, which mimics the blood vessel surrounding the solid tumor, is the generation of microvasculature. The blood circulation system is essential in many cell processes and many pathologies including cancer and modulate drug sensitivity [94]. The effect of chemotherapeutics agents on cancer cells has been studied previously using microfluidic technology. Wong et al. [94] reported the construction of cancer cells on-chip from cancer cell lines and cells derived from primary tumors, and tested against cisplatin, bortezomib, vorinostat, and epirubicin. 3D microfluidic chip-based *in vitro* model was also developed to evaluate the viability of cancer cells in 3D matrix in response to gemcitabine at different concentrations [61]. The microvasculature was seen in CRC-on-a-chip 3D model and the cell death demonstrated in a gradient manner following the gemcitabine exposure [61]. Overall, these key features in microfluidics-based model have set the stage for physiologically relevant *in vitro* models to better predict anti-cancer drug response.

Nevertheless, microfluidics-based cultures present with significant drawbacks in meeting the needs of *in vivo* microenvironment which suggest that it still has a long way to realize its fully usage in anti-cancer drug screening. The particular key features mimicked in the microfluidics-based model such as shear stress and microvasculature are not fully functional and thus led to transient effects of cell-drug behavior and inaccurate drug readout [18]. The *in vitro* model as a tissue-on-a-chip which is highly expensive also do not exactly capture the important essence of TME with abnormal and sprouting blood vessels network, and heterogenous cell types [95, 96]. In conclusion, more technical optimization in microfluidic technology is needed for higher physiological relevance model in drug development and testing.

### 3D culture

Research on the molecular pathways underlying cancer progression is highly complex in nature mainly due to significant heterogeneity observed among tumors. Despite being widely used in pre-clinical study to investigate molecular profiling of cancer and screening of anti-cancer drugs, cell line establishment is laborious, inefficient, and time-consuming. Only selected uncommon clones have adapted to respective culture conditions, therefore they are unable to replicate tumor variety thus making them unsuitable model to study tumor heterogeneity [2, 97]. The lack of cell-cell interactions in a 3D environment and the 2D nature of cancer cell lines grown in culture have also limited their ability to accurately replicate and sustain the genetic complexities observed in physiological condition. Additionally, the representation of tumor heterogeneity *in vivo* remains unmet in 2D culture conditions as the poor simulation of original tumor niche in which tumor mass is grown three-dimensionally. Moreover, it is difficult to utilize 2D culture for high-throughput analysis [3, 98].

Owing to the major drawbacks of 2D cell culture models in drug screening has put on emphasis on the importance of effective and solid recapitulation of tumor biology, as well as the complex and heterogeneous nature of TME. These important aspects are highlighted in 3D cell culture system thus providing an improved clinically relevant model in predicting drug response.

The earliest success story of developing organoids from ASCs was reported by Clevers et al. [99] driven by discoveries that leucine-rich repeat-containing G protein-coupled receptor 5 (LGR5) as promoter for Wnt signalling in adult intestinal stem cells. Sato et al. [100] developed the first 3D culture of epithelial cells from a single LGR5^+^ stem cell, which are embedded into Matrigel and maintained using serum-free medium containing growth factors such as R-spondin 1 (a Wnt agonist and LGR5 ligand), epidermal growth factor (EGF), and the bone morphogenetic protein (BMP) antagonist, noggin. This culture protocol has been adopted by many researchers ever since to develop organoids for human and mouse epithelial cells.

**Figure 2:**
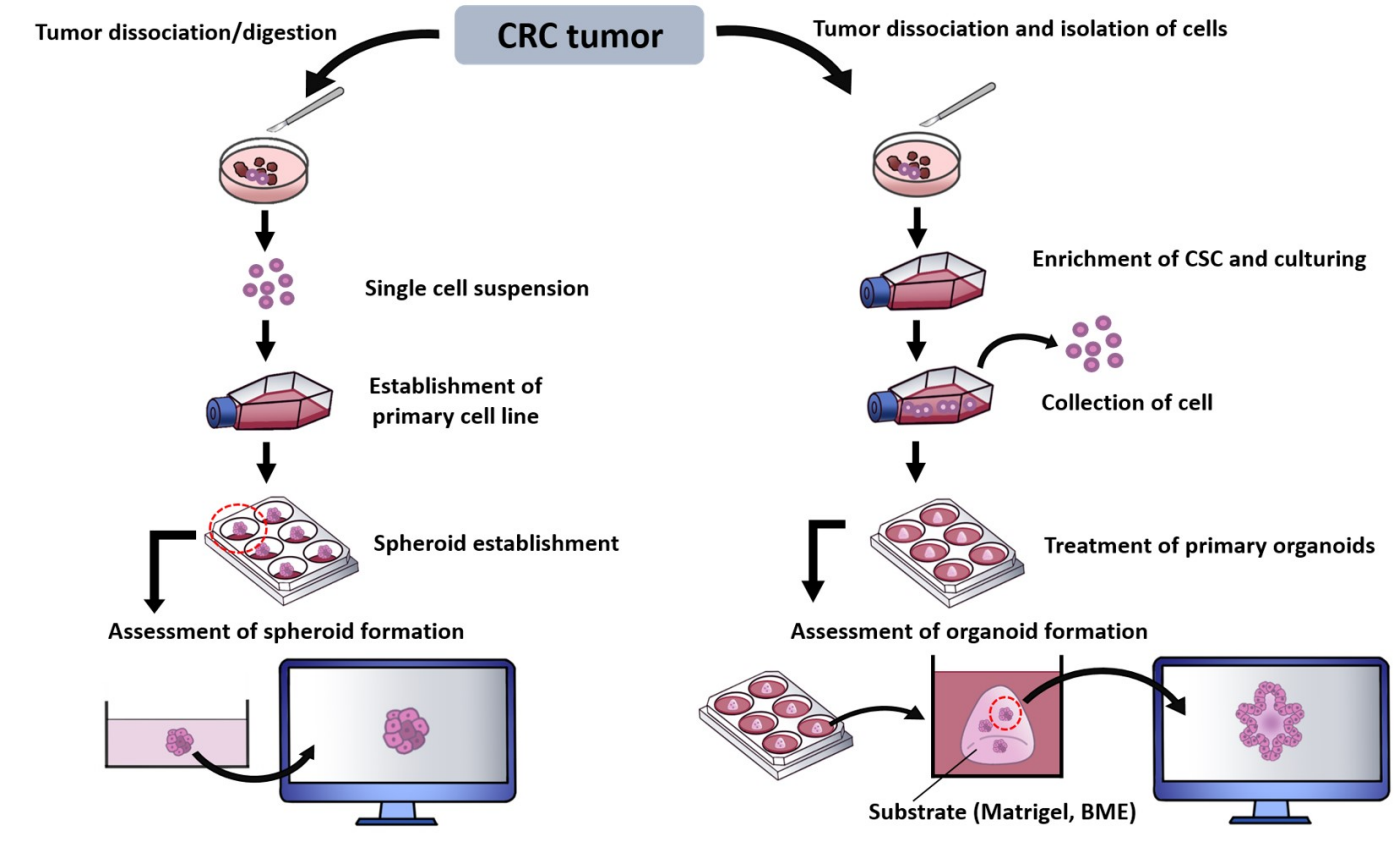
Summary of methods in establishing CRC spheroids and organoids. BME: basement membrane extract; CSC: cancer stem cell

Laboratory techniques have been introduced to generate 3D culture models to allow in depth study of multifaceted features of a tumor niche. These establishment of spheroids and organoids from CRC sample can be represented as in Figure 2 [30, 101–104].

It is imperative that the construct of both spheroids and organoids ideally meet the native CRC characteristics for efficient and reliable drug screening. Thereby, several 3D culture methods have been developed by producing spheroids and organoids and strategically improved towards mimicking the CRC. Generally, spheroids are generated by using conventional culture media supplemented with essential factors for the continual growth of the spheroids. It has been shown that spheroids retain patient sensitivity to treatment and the success rate of such replicability is dependent on the viability of spheroids [105].

Broadly, 3D culture methods can be classified into scaffold-based consisting of Matrigel or BME embedding, air-liquid interfaces (ALI), and scaffold-free systems. This classification is determined by the presence or absence of a gel substrate and the specific technique used to embed the cells [106, 107]. The inclusion of various growth factors dedicated to different tissues and co-cultivation with immune cells are highlighted to replicate the TME, thereby emulating the matrix conditions for 3D culture [100].

The later system; scaffold-free with hanging drop, liquid-overlay, and microfluidic-based methods are developed for spheroids production. These methods provide the platform to mimic the TME key features such as oxygen gradients and hypoxia that are relatively important for high-throughput drug screening [62]. The multiple layers formation of the spheroid retains the avascular characteristic of tumors with an engineered construct of passive diffusion that can result in hypoxia. These methods however pose disadvantages, as listed in Table 1.

To date, the key method of growing organoids derived from GI tract is the use of Matrigel. Matrigel or BME, purified from Engelbreth-Holm-Swarm (EHS) mouse sarcoma line is considered the ‘gold standard’ reconstituted ECM protein mixture and substrate used in establishing 3D culture. The applications of Matrigel over the past decade have greatly surpassed other biomaterials, for its property in supporting organoid growth. Matrigel is composed of 60% laminin, 30% type IV collagen, 5% nidogen, 3% heparin sulfate proteoglycan, and 1% entactin [108] as well as repertoire of growth factors such as EGF, TGF-β, insulin growth factor 1 (IGF-1), and basic fibroblast growth factor (FGF) [11]. Technically, organoids in Matrigel are often coupled with endpoint chemosensitivity assays such as live-dead staining for drug readout. In addition to that, an imaging system, high-speed live-cell interferometry (HSCLI) is recently introduced, capable of capturing a clear geometric distribution of 3D organoids in Matrigel and assessing heterogeneous treatment responses akin to native tumors [109]. Despite widely use, this commercialized matrix has several drawbacks. Due to its origin, it is presented with large batch-to-batch variation in cultured organoids. There is also a potential risk of transmission of animal pathogen due to its origin [110] and inability of tumor ECM-based Matrigel to recapitulate tissue-specific microenvironment GI for organoids. In addition, Matrigel is not easily manipulated to produce organoid niches for specific organs thereby limiting maturation cell-responsiveness to drugs [111]. Thus, alternatives to Matrigel have been considered in developing 3D organoids including synthetic hydrogels such as synthetic poly(ethylene glycol) (PEG) hydrogels modified with ECM peptides and protease-degradable peptides or natural hydrogels [112, 113]. Gelatin-phenol (gelatin-Ph) and hyaluronic acid-phenol (HA-Ph) hydrogels that are covalently crosslinked are introduced with tailorable mechanical properties for optimal organoid culture and drug screening purpose. Ng et al. [67] demonstrated that by chemically modifying the hydrogels with different ranges of mechanical stiffness to mimic the ECM stiffness, the hydrogels supported the organoid growth and sensitivity to CRC therapeutic drugs compared to Geltrex, an animal-derived matrix equivalent to Matrigel.

Organoid composition is commonly evaluated through immunofluorescence and immunohistochemical imaging using sections or whole mount. The staining of specific cell marker’s antibody further determines various cell types’ spatial distribution and proportion [114].

The first organoid model with a defined genetic setup was developed from pancreatic, gastric, and colonic tissue of mice by utilizing the ALI method. Li et al. [115] reported the development of dysplasia in pancreatic and gastric organoids resulting from G12D mutation in Kirsten rat sarcoma (*Kras*^G12D^), loss of *p53* loss or both, and formation of adenocarcinoma post *in vivo* transplantation. Conversely, organoids from colon combination required mutations of adenomatous polyposis coli (*Apc*), *p53*, *Kras*^G12D^ and *SMAD4* for progressive transformation to form invasive adenocarcinoma-like histology *in vitro* and tumor formation *in vivo* which recapitulate multi-hit models of CRC, in comparison to the more promiscuous transformation of organoids from small intestines [115].

Established 3D cultures from patient also known as PDOs are used to address the deficiency implicated in conventional preclinical models [116]. In addition, organoids possess the ability to efficiently produce *in vitro* disease models at minimal cost in comparison to animal models [66, 117]. Organoids or spheroids have enabled better reconstruction of a tumor, and this can be further manipulated by co-culture method [118]. The application of spheroids and organoids in drug screening for CRC is elaborated in the following sections.

## Application of spheroids and organoids for drug screening

The lack of representative tumor *in vitro* model as such CRC with its complex and heterogeneous architecture hinders its full utility in providing a clinically informed decision making and novel therapeutic strategies. The advents of 3D cell culture via construct of spheroids and organoids have emerged as improved tumor model, bridging the gap between the *in vitro* and *in vivo* studies. Following that, the capacity of spheroids and organoids for robust drug screening has been discussed to a certain extent for clinically relevant application. The following section will describe the latest applications of spheroids and organoids in drug screening and discovery for CRC.

### CRC spheroids for drug screening

3D tumor spheroid is developed by dissociation of tumor tissues or cancer cells. It was commonly applied to study CRC model where several 3D spheroid models have been established from CRC cell lines such as DLD-1, HCT116, and LuM1, patient-derived xenografts (PDX), and patient-derived cells for drug screening [119–122]. Some studies have pointed out that 3D spheroid models produced similar treatment response to *in vivo* and clinical studies [101, 123].

Using SW620 tumor spheroids grown on microfluidic tumor-on-chip platform, the combination of 7-ethyl-10-hydroxycamptothecin [SN38; the active metabolite of the Topoisomerase I (TOP1) inhibitor-irinotecan] and AZD0156 potentiate DNA double strand breaks and induced cell death and reduced proliferation of the cells [124]. This platform can be used to predict drug pharmacokinetics, pharmacodynamics, and efficacy *in vivo*.

MCTS have been developed by culturing different cell types such as fibroblasts, endothelial cells, and immune cells to better mimic the native heterogenous and multifaceted TME as their considerable modulation of chemoresistance in particularly CRC [64, 125]. It was highlighted that 3D co-culture spheroids of pancreatic ductal adenocarcinoma (PDAC) cells and stromal cells namely CAFs resulted in greater loss of gemcitabine potency than 2D co-culture model [87]. The spheroids of co-culture CRC cell line with normal fibroblast line, CCD18Co also displayed chemoresistance behavior wherein the cancer cells were observed with improved cell metabolic activities in the presence of 5-FU, regorafenib, and erlotinib [63]. Interestingly, another CRC spheroids of co-culture between CRC cell line, LS174T cells and CAFs were developed for potential therapeutic testing [64]. The co-culture CRC spheroids showed great sensitivity to phosphatidylinositol 3-kinase (PI3K) inhibitor, LY294002 with dramatic decline of tumor growth [64]. The developed 3D triculture CRC spheroid model constituted of HCT116 cells, human intestinal fibroblasts, and primary monocytes which transdifferentiated into macrophages was tested for Nutlin 3a (Nut3a) and compared with 2D models [69]. The 3D spheroid model showed an anti-proliferative effect but not at a higher extent as shown in 2D model. Overall, it is worth noting that 3D spheroid models produced a different phenotype in its drug sensitivity when compared to 2D models. Whilst, with respect to its *in vivo* counterparts, 3D spheroid models of both monoculture and co-culture produced quite similar phenotypes [64].

Accumulating studies have highlighted the greater chemoresistance in 3D spheroid models in comparison to 2D as observed in CRC and other carcinomas [23, 64, 87, 126, 127]. This might be explained by the multiple cell layers in the dense spheroid model wherein the cells in the outer layer act as physical barriers, limiting the drug penetration. The presence of these physical barriers can lead to hypoxia and acidosis, which are associated with chemoresistance [128]. The hypoxic environment especially cells in the inner layer can trigger release of hypoxia-inducing factors (HIFs) in the cancer cells. Additionally, the acidity environment affects the drug permeability to cancer cells and thus weakly acidic drugs have higher permeability and response [129]. In contrast, some tumor spheroid with enhanced sensitivity displayed a distinct cell morphology in comparison to 2D model. Some spheroids displayed irregular shape with less sphericity and scattering outgrowth. The decreased sphericity allows for appropriate oxygen and nutrient retention to maintain the function of multilayered cells [130]. In Dolznig et al. [64], their 3D CRC spheroid model showed organized glandular structures with mucin production. Another CRC spheroids of HT29/fibroblast were observed with elevated fibronectin expression which coincide with the significant loss of doxorubicin potency [60].

Indeed, tumor spheroid with multilayered approach enable the recapitulation of native TME. By culturing different cell types such as fibroblasts and tumor-associated macrophages (TAMs) can provide further clarity into their roles on the malignant progression and chemoresistance unfeasible in traditional 2D models. However, similar to microfluidics-based cultures, it remains challenging to determine the individual cell effect in the tumor spheroid upon treatment. Furthermore, the avascular characteristic with passive chemical (e.g., nutrient and oxygen) diffusion may perturb the cell function, especially the cells in the inner layer of spheroid and thus risk of misleading results in predicting drug response. Given these limitations, the spheroid model is concurrently used only in small cohorts and as supplementary to *in vivo* studies for preclinical drug testing [23]. Nevertheless, spheroid enables precise characterization of chemotherapies and potential drug efficacy with its increasing heterogeneity that is unfeasible in other *in vitro* models, representing an improved model for preclinical drug testing.

### CRC organoids for drug screening

Sato et al. [89] have successfully optimized culture systems and reported long-term expansion of epithelial organoids from colon of mice and small intestine and colon of human which enable the inflammation and neoplastic changes in GI tract to be studied. They further found that replicative potential of ASCs *ex vivo* was not restricted based on study related to these cultures. Mout et al. [131] reported on organoids production from 35% of collected patient samples with metastatic prostate cancer whereby one long-term culture harbor comparable drug resistance as to that of primary tumor. Majority of organoids could only be maintained in culture between 6–8 weeks.

Organoids can be successfully constructed from both normal and cancer human colonic tissue with its mimicking of genotypic and phenotypic heterogeneity to CRC [132, 133]. Organoids have enabled more accurate prediction of clinically relevant drug response and improve CRC patient survival with tailored personalized treatment and novel drug discovery.

A living 3D culture biobank of CRC was established by van de Wetering et al. [16] consisting of 22 tumor organoids and 19 normal adjacent organoids, isolated from 20 patients. Majority of organoids are shown to preserve the genomic characteristics with consistent mutation patterns in CRC. “Cystic and solid” organization of the epithelium of primary tumors was also preserved in the organoids as shown by the hematoxylin and eosin (H&E) staining. Transcriptomic profiling of the organoids reveals subtle differences in their genome highlighting the heterogeneous characteristic. These results showed that organoids recapitulated several properties of the original tumors from which they derive and thus can be relevantly applied for high-throughput drug screening and detection of gene-drug associations. For example, CRC organoids with *Kras* mutation are resistant to the combination targeted inhibitors of EGF receptor (EGFR) and mitogen-activated protein kinase (MEK) [134] but showed promising viability inhibition upon tested with new combinations of trametinib, neratinib, and trastuzumab [135].

Conventional organoid-related work mainly involved bulk sample is devoid of cell-type diversity and heterogeneity [16, 136]. Henceforth, the new advent in single cell technology namely single-cell RNA-seq has been introduced, allowing the investigation focusing on the tumor composition and functional heterogeneity [137].

Wang et al. [2] conducted single-cell RNA-seq on PDOs, normal colon, and CRC tissues from seven patients. It was reported that CRC organoids faithfully maintained gene expression profiles of cancer cells whereas organoids derived from normal tissues showed tumor-like characteristics at the whole transcriptome level although still retained normal molecular features including copy number variations (CNVs), genomic mutations, and normal DNA methylation patterns. This includes the portrayal of epithelial-mesenchymal transition (EMT) phenotype in organoids based on the co-expression of vimentin (VIM) and epithelial cellular adhesion molecule (EPCAM).

In addition, scientists have also explored the prospect of using analyte from liquid biopsy such as CTCs in developing organoids which represent different pathological conditions [138, 139]. Characterization of CTCs provide insight into mechanism of metastasis thus exploiting CTCs can prevent and treat metastatic cancer [140]. Moreover, CTC-derived xenograft (CDX) models and CTC-derived *ex vivo* cultures have been utilized as tractable systems to study tumor-initiating cells (TICs) and discover more treatment options [141]. One of the earliest and most successful attempts to develop long-term culture of CTCs isolated from patients is done by Gao et al. [142]. They employed organoids from prostate cancer which mimic tumor-specific characteristics. There was no change in gene expression seen between the primary tumor and CTC line. The capacity of CTC to develop tumors was confirmed using a mouse model.

De Angelis et al. [68] developed CTCDOs from an orthotopic CRC xenograft model and subjected them to proteomic analysis, immunohistochemistry, immunofluorescence, flow cytometry, tumor-forming capacity, and drug screening analyses. CTCDOs showed a hybrid EMT state and elevated expression of stemness-associated markers such as two homeobox transcription factors; Goosecoid and pancreatic duodenal homeobox gene-1 (PDX1) that were also found in CTCs of CRC patients. CTCDOs showed a greater migration and invasion and responded differently to pathway-targeted drugs in comparison to xenograft-derived organoids (XDOs). CTCDOs displayed higher sensitivity than XDOs towards drugs affecting the Survivin pathway. Survivin and X-linked inhibitor of apoptosis protein (XIAP) levels were decreased in CTCDOs which further induce CTCDOs death. These results are indicative of CTCs-CRC characteristics and can be applied for the identification of new prognostic biomarkers of CRC and serve as a basis to design potential therapeutic strategies, especially for mCRC.

On top of CRC, organoids have also been used as a model to study other GI entities as they can be isolated from all GI tissues including esophageal, gastric, liver, pancreatic, small intestinal and colorectal tissues, and cancers of these tissues [143]. He et al. [144] has successfully developed organoids derived from mCRC patients which able to maintain genetic and phenotypic heterogeneity of tumors of origin. Drug sensitivity assays were performed on these PDOs and half-maximal inhibitory concentration (IC_50_) values of 5-FU, oxaliplatin, and irinotecan were obtained. This *in vitro* analysis proved the potential of PDOs in assessing response to chemotherapeutic drugs and predicting survival of mCRC patients.

Compared to the above pre-existing *in vitro* methods, organoids are considered to be a superior model for complex diseases such as cancer and effectively broadens the capacity for high throughput drug screening. The establishment of organoids has been extensive catering to the inadequacies of other preclinical models, hence the construct of them ideally focuses on the representation of native tumor characteristics with heterogeneity [132]. Tumor heterogeneity constitute an important aspect towards the development of therapeutic failure by chemoresistance [145]. Understanding tumor heterogeneity through tumor organoids can possibly reveal further insights on the biological significance related to drug response and help in prediction of drug efficacy and resistance. PDO of 12 patients were developed and subjected to sequencing which revealed various drug responses indicative of mutational intratumor heterogeneity of CRC [69]. Another study exhibited various drug responses upon tested on each single cell-derived organoid which were identified to express different oncogenic mutations in especially cancer driver genes including MutL homolog 1 (*MLH1*), erb-b2 receptor tyrosine kinase 2 (*ERBB2*), and SRY-box transcription factor 9 (*SOX9*) [70]. The recurrent mutations in the primary CRCs were shown recapitulated in the organoids of both studies enabling functional integration of transcriptomic heterogeneity in correspondence to the heterogeneous drug responses in CRC.

Organoid also allows more precise profiling of CRC thus driving drug screening, more effectively cater to subpopulation of patients. Yan et al. [146] revealed key mutational and transcriptomic alterations in microsatellite stable sporadic early-onset CRC (EOCRC). CRC PDOs also have been utilized as a genetic model for investigation of the function of driver genes. Notably, the use of PDO has been exclusively designed to represent diverse genomic and proteomic characteristics from different patients, unleashing the possibility in tailoring personalized therapy. The whole-exome sequencing on multifocal CRC organoids was performed which demonstrated intertumoral heterogeneity between patients [147]. The significant similarity and stability of organoids genetic and epigenetic to tumor derived from patient has led to the establishment of living biobank of CRC organoids [16, 148]. Further, the recapitulation of primary tumor heterogeneity by the organoids were also achieved in the perspective of its histological and cellular composition. Most organoids developed by Kim et al. [69] and Okamoto et al. [149] contain lumen and different clusters of cell type such as stem-like and more proliferating cell clusters respectively which resemble the isolated primary CRCs. The unlimited capacity for proliferation in the organoids were shown while still retaining the stem cell hierarchies with differentiation that resembles primary tumors, unlike the 2D cultures after long serial passaging [11, 89]. This characteristic of organoid allows accurate assessment in drug testing and the chemoresistance related mechanism. For example, the chemoresistant capacity with the proportion of Wnt-CRC cells remained unchanged in the organoids upon tested with 5-FU and oxaliplatin, different to that of spheroids after serial passaging [150]. Boos et al. [151] also showed that CRC cells in PDOs following prolonged exposure to combination of folinic acid, 5-FU and irinotecan (FOLFIRI) were less sensitive to dual pathway of anti-EGFR. The acquired chemoresistance in the organoids was associated with the augmented cellular- myelocytomatosis oncogene (*c-MYC*) and *c-MYC* related genes which lead to suggestion of potential strategy to circumvent anti-EGFR therapy resistance as also demonstrated in Elbadawy et al. [152]. Taken together, organoid constitutes an improved tumor model with the recapitulation of tumor heterogeneity and testing drug responses in long-term cultures.

Furthermore, organoids have been suggestively to hone its capacity in modelling different cancer subtypes with the help of genetic engineering technology. Serrated CRC, for example, represents an aggressive subtype of CRC with often relative resistance to anti-EGFR therapy and poor prognosis [73]. The organoids with clustered regularly interspaced short palindromic repeats-Cas9 (CRISPR-Cas9) which incorporate common genetic alterations [*Kras* or B-Raf proto-oncogene (*BRAF*)] revealed a specific tumor niche and prognostic gene markers representing human serrated CRC [74, 153]. Such modelling can benefit drug response characterization and chemoresistance study. The serrated CRC organoids with *Kras* or *BRAF* mutations and in combination with R-spondin fusions have highlighted the mechanism related to Wnt inhibition that may represent the acquired chemoresistance in serrated CRC [75, 76]. Another serrated CRC organoids driven by mutations in *Kras* and codon 600 of exon 15 of *BRAF* gene (*BRAF V600E*) mutations revealed the activation signaling of TGF-β that is associated with increased chemoresistance [77, 154].

3D culture also is a strong modality to study multi-omics characterisation of cancer which provide insight into carcinogenesis process. Previous study by Della Chiara et al. [155] has reported the development and maintenance of PDOs from CRC for transcriptomic and epigenomic studies. They have discovered that transcriptional activators namely Yes-associated protein (YAP)/transcriptional co-activator with PDZ-binding motif (TAZ)’s role in sustaining a core gene-regulatory network of CRC which is vital for the maintenance of the neoplastic cell state. This may serve as a potential target for drug development.

## Challenges of spheroid and organoid technology

One of the greatest hurdles of spheroid and organoid technology is the inability to recreate the microenvironment *in vitro*. It is thought that not all organoids were exactly the recapitulation of primary tumors with their distinctive drug responses [132]. For example, in chemoresistance studies where organoids predictive clinical utility is limited to certain drug combinations. The CRC organoids in Ooft et al. [72] did not reproduce the patient outcome to combination of 5-FU and oxaliplatin. This is consistent with Narasimhan study [71] that showed failed recapitulation and association with patient response to oxaliplatin-based therapy. PDOs and the inclusion of TME components have been considered to bypass the issue.

It is also worth noting that reproducibility of spheroids and organoids is vastly challenging as their production is a highly complex process and depends on various factors such as cell type, cellular state, and growth rate [156]. For example, spheroid production is hindered by the lack of size uniformity where spheroid size can range between 65 µm and 300 µm when generated by spinner flasks [157–160]. It has been proposed that microfabrics and microscale platforms can be applied to overcome some of the limitations in spheroid and organoid production.

The functionality and maturation of both spheroids and organoids as pre-clinical models and in drug testing is limited as the formation of tissue necrosis in their inner layer. This mainly impacts the large-size spheroids and organoids with their avascular characteristics and reduced oxygen and nutrient diffusion. Cells in the inner layer of spheroids and organoids suffer from the lack of a functional vascular network and poor perfusion flow, hence the necrosis [161]. Attempts have been made to bypass the issue including the use of an economical 3D bioprinting technology for higher physiological models. Compared with organoids built on conventional culture method, 3D bioprinted organoids are shown to recapitulate better complex cell physiology with signaling molecule carriers and provide precise gene-drug association [162]. The incorporation of microfluidics technology has also helped to deliver nutrients efficiently via organ-on-a-chip devices [163].

Additionally, among the difficulties in establishing spheroids and organoids are the use of various growth factors as well as the application of laborious techniques that were involved to sustain the optimum growth of these structures. A repertoire of growth factors such as Noggin and EGF were included in the culture medium formulation along with scaffold namely Matrigel [155]. This subsequently led to a higher cost in conducting experiments using 3D structure, particularly organoids, compared to conventional 2D monolayers. Moreover, the lengthy process of 3D culture establishment serves as one of the challenges in study involving these entities. Analysis of the spheroid and organoid which often require immunofluorescence staining may act as a disadvantage to the institutions experiencing lack of robust instrument.

Expensive cost of developing organoids has propelled scientist to find an alternative way in reducing costs. For instance, conditioned medium of selected cells which can be produced as much lower cost, has been tested to be used in co-culture experiment, to mimic the microenvironment of organ of choice [164]. Technical aspect in developing organoid also limits its application whereby this technique presented with low batch-to-batch reproducibility and expensive cost. The CTCDOs are deemed complicated due to its technical difficulties, and long-time taken in growing cultures. These factors could contribute to its incompatibility in informed clinical decision-making [165]. To overcome these challenges, a standardization in the protocol has to be applied to ensure reproducible organoid growth in culture at a more efficient cost for drug screening study [166]. The inclusion of multi-parametric validation such as transcriptomic profiling would help generate a more robust drug screening platform [167]. More reliable and functional endpoint assays are needed for improved clinical fidelity of both spheroids and organoids.

As stated in the previous section, spheroid and organoid technologies are limited by the lack of evidence in physiological condition. PDO xenograft (PDOX) which is a 3D *in vivo* model has been developed in recent years and used as continuum for *in vitro* model. Following the initial characterisation, focused or high-throughput screenings of individual drugs or drug combinations can be conducted in a repetitive manner to identify potential treatment approaches [168]. These approaches are then subjected to additional evaluation using individualized *in vivo* models.

Mao et al. [169] reported on a technique which combines computational predictions with rigorous *in vitro* and *in vivo* validations to improve the discovery of prospective treatment agents for CRC including trametinib, bortezomib, and fedratinib. In addition, Jian et al. [170] innovated a PDOX-liver metastasis (PDOX-LM) technique transplants human CRC organoids into mouse spleens to establish a xenografted liver metastases model. These *in vivo* metastatic models imitate CRC metastases better than subcutaneously generated models [171–173] and improve medication effectiveness evaluation [174, 175]. The establishment of PDOX expands the functionality of PDO and potentially addresses the shortcomings of both PDX and PDO. Still, organoids are foreseen as robust *in vitro* preclinical model with improved physiological relevant to complex and heterogeneous cancer such as CRC and thus reveal clinical translatability in anti-cancer drug testing.

## Conclusions

Spheroids and organoids have been recognized as versatile *in vitro* preclinical models and tools for translatable and reliable clinical outcomes in comparison to other *in vitro* methods for anticancer drug screening. However, current work showed that these 3D cultures are still somewhat far from being implemented into clinical practice and testing for precision medicine. The advancement in the establishment of spheroids and organoids including co-culture with various cellular components of colorectal TME, addressing the intra and intertumor heterogeneity of CRC and improvement in spheroid and organoid development laboratory technique may greatly enhance the reproducibility of the 3D culture. Combined use of spheroids and organoids will also reduce the unnecessary animal testing and subsequently the cost and time for the analysis. Improved 3D culture systems also will ensure the robustness of drug testing for CRC and offer vast opportunities to design treatment strategies through the application of spheroid and organoid technology.

## Abbreviations

2D: two-dimensional
3D: three-dimensional
5-FU: 5-fluorouracil
ASCs: adult stem cells
BME: basement membrane extract
BRAF: B-Raf proto-oncogene
CAFs: cancer-associated fibroblasts
CRC: colorectal cancer
CTCDO: CTCs-derived organoid
CTCs: circulating tumor cells
ECM: extracellular matrix
EGF: epidermal growth factor
EGFR: epidermal growth factor receptor
EMT: epithelial-mesenchymal transition
GI: gastrointestinal
Kras: Kirsten rat sarcoma
Kras^G12D^: G12D mutation in Kirsten rat sarcoma
LGR5: leucine-rich repeat-containing G protein-coupled receptor 5
mCRC: metastatic colorectal cancer
MCTS: multicellular tumor spheroids
PDO: patient-derived organoid
PDOX: patient-derived organoid xenograft
TGF-β: transforming growth factor beta
TME: tumor microenvironment
Wnt: wingless/integrated

## Declarations

### Copyright

© The Author(s) 2024.
